# Co-administration of Naringin and NLRP3 Inhibitor Improves Myelin Repair and Mitigates Oxidative Stress in Cuprizone-Induced Demyelination Model

**DOI:** 10.2174/1570159X23666241206102022

**Published:** 2024-12-06

**Authors:** Fatemeh Kalaki-Jouybari, Moein Shirzad, Mohammad Javan, Maryam Ghasemi-Kasman, Mehdi Pouramir

**Affiliations:** 1 Student Research Committee, Babol University of Medical Sciences, Babol, Iran;; 2 Department of Clinical Biochemistry, Faculty of Medicine, Babol University of Medical Sciences, Babol, Iran;; 3 Department of Physiology, Faculty of Medical Sciences, Tarbiat Modares University, Tehran, Iran;; 4 Cellular and Molecular Biology Research Center, Health Research Institute, Babol University of Medical Sciences, Babol, Iran;; 5 Department of Physiology, Faculty of Medicine, Babol University of Medical Sciences, Babol, Iran

**Keywords:** Naringin, MCC950, Cuprizone, Oxidative stress, Demyelination, Inflammation

## Abstract

**Background:**

Naringin and MCC950 as an inflammasome inhibitor have exhibited numerous pharmacological activities, including antioxidant and anti-inflammatory effects. The present study has examined the combined impacts of naringin and MCC950 on the levels of oxidative stress, demyelination, and inflammation in the cuprizone (CPZ)-induced demyelination model.

**Methods:**

In order to induce demyelination, CPZ (0.2% w/w) was added to the normal diet of mice for 42 days. Subsequently, the male C57BL/6 mice received naringin (oral administration), MCC950 (intraperitoneal injection), or their combination for 14 days. Working memory was tested by the Y maze. FluoroMyelin staining, MOG, and GFAP immunostaining assessed the demyelination extent, myelin intensity, and astrocyte activation, respectively. Oxidant/antioxidant biomarkers were measured using colorimetric techniques. The expression levels of *MBP*, *PDGFRα*, *Olig2*, *Nrf2*, *HO-1*, *NQO-1*, *GSK3β*, *IL1β*, and *IL18* were assessed by reverse transcription-quantitative polymerase chain reaction (RT-qPCR).

**Results:**

Our results indicated that the co-administration of naringin and MCC950 improved working memory and antioxidant capacity. A significant reduction was found in the extent of demyelination and inflammatory mediatorsin naringin and MCC950-treated mice. In addition, co-administration of naringin and MCC950 elevated the expression levels of pro-myelinating and antioxidant markers.

**Conclusion:**

These findings indicated improvement of the working memory through co-administration of naringin and MCC950, which might be partly mediated by enhancing antioxidant capacity, promoting remyelination, and mitigating inflammation in the CPZ-induced demyelination model.

## 1 INTRODUCTION

One of the neuroinflammatory disorders that affects the central nervous system (CNS) is multiple sclerosis (MS) [1]. The pathogenesis of MS is significantly influenced by the involvement of neuroinflammation, oxidative stress, activated microglia, and myelin sheath damage [2]. Despite the existence of numerous disease-modifying therapies, there are no effective pharmaceutical interventions that can impede the inexorable progression of the disease [3]. Thus, it is imperative to conduct further investigation into alternative therapies and the pathogenic factors implicated in the neurodegeneration as well as demyelination associated with MS.

Neuroinflammation involves the activation of innate immune cells, especially microglia. Upon activation, microglia stimulate protein oligomers known as inflammasomes. The most distinct inflammasome within the CNS among the NOD-like receptor (NLR) family is recognizable as the NLR family pyrin domain containing 3 (NLRP3). The NLRP3 activation elevates the levels of two pro-inflammatory cytokines (IL-1β and IL-18) [4]. Furthermore, the active forms of these cytokines exhibit a remarkable rise in MS patients [4]. A selective NLRP3 inflammasome inhibitor molecule, called MCC950, significantly reduces motor defects in MS animal models [5]. MCC950 blocked the production of IL-1β and IL-18, microglia secretion, as well as diminished neuroinflammation [6].

Reactive oxygen species (ROS) are generated from organelles with high levels of oxygen consumption, particularly in mitochondria [7]. The perturbation in the equilibrium of the generation and accumulation of ROS leads to the activation of NLRP3 inflammasome and subsequent inflammation reactions while also instigating oxidative stress [7]. The nuclear factor erythroid 2-related factor (*Nrf2*) is a factor primarily responsible for the transcription of antioxidants and plays a role in preserving oxidative stress homeostasis by controlling the protective genes expression, such as heme oxygenase-1 (*HO-1*), nicotinamide adenine dinucleotide phosphate (*NADPH*), and quinone reductase-1 (*NQO-1*) [8]. Furthermore, the antioxidant activity of the body has a relationship to the presence of enzymes that effectively counteract and disintegrate ROS generation, such as superoxide dismutase (SOD), glutathione peroxidase (GPx), and catalase (CAT) [9]. Various regions of MS patient brains, such as the corpus callosum, upregulate glycogen synthase kinase-3β (*GSK-3β*) expression [10]. The active form of *GSK-3β* inhibits the expression of transcription factors, such as *Nrf2*, thereby averting the inhibition of oxidative stress and inflammation [11].

Naringin (4',5,7-trihydroxyflavanone-7-rhamnoglucoside) with a flavanone glycoside structure is recognized as the predominant flavonoid in citrus fruits, particularly grapefruit [12]. A pivotal attribute of this food polyphenol is its ability to cross the blood-brain barrier (BBB) [13]. Naringin displays promising therapeutic effects, notably anti-inflammatory and antioxidant activities in various neurological disorders [13, 14]. It has been shown that naringin upregulates the *Nrf2* gene expression to counteract the toxic impact of free radicals. Furthermore, this flavonoid exerts a regulatory influence on the expression of antioxidant enzyme transcripts [15, 16].

Cuprizone (CPZ), as a copper chelator, operates primarily through mitochondrial disruption, resulting in various demyelination of brain regions, particularly the corpus callosum [17]. Astrogliosis, exacerbation of microglial activation, and cognitive impairments have been found in the CPZ model similar to those observed in MS patients [18].

The present study was designed to evaluate the impacts of the co-administration of naringin and MCC950 on the working memory of mice with CPZ-induced demyelination. Then, the extent of demyelination, oxidative stress level, inflammation, and remyelination was examined in the corpus callosum of mice.

## 2 MATERIALS AND METHODS

### 2.1 Chemicals and Reagents

CPZ, MCC950, and naringin were purchased from Sigma-Aldrich (St. Louis, MO, USA). To achieve a concentration of 0.2% (w/w), CPZ was blended into standard mouse chow. The mixture was then formed as standard pellets, which were fed to the mice for a period of 6 weeks [19]. Naringin was dissolved in a vehicle consisting of 5% Tween in saline. Phosphate-buffered saline (PBS) was used as a solvent for MCC950. The primary antibodies, including GFAP (Z0334) and MOG (MAB5680), were obtained from Dako and Millipore companies, respectively. Secondary antibodies, goat Anti-Rabbit IgG H&L (Alexa Fluor^®^ 488) (ab150077) and goat anti-mouse IgG H&L (Alexa Fluor^®^ 594) (ab150116) were obtained from Abcam. DAPI and FluoroMyelin were purchased from Sigma-Aldrich and Thermo Fisher, respectively. The relevant primers and biochemical kits were supplied by the Pishgam and Kiazist companies (Iran), respectively. The chemical structure of naringin is shown in supplementary Fig. (**S1**).

### 2.2 Animals

Forty male C57BL/6 mice (6-8 weeks, weighed between 20-25 g) were used in the study. The mice were purchased from Pasteur North Research Institute in Amol (Iran). Animals were housed in groups of 4 per cage and maintained in a temperature-controlled (25 ± 2°C) with a 12-hour/ 12-hour light and dark cycle. Also, food and water were accessible freely. The humidity was controlled within 55-60%. The Ethics Committee of Babol University of Medical Sciences approved all procedures (Approval No: IR.MUBABOL.REC.1401.08.03).

### 2.3 Experimental Design

Forty mice were randomly divided into five groups (n=8) as follows:

- Group 1 (control group): without any intervention
- Group 2 (CPZ): Mice received the chow containing 0.2% CPZ to induce demyelination, and then saline+ 5% Tween was orally administrated as the vehicle of naringin.
- Group 3 (CPZ+naringin): CPZ was added into the normal chow for 6 weeks, and then, naringin at a dose of 50 mg/kg was administrated *via* oral gavage for 2 weeks [20].
- Group 4 (CPZ+MCC950): Mice were treated with intraperitoneal (i.p.) injections of MCC950 at a dose of 5 mg/kg every other day after receiving CPZ for 6 weeks [21, 22].
- Group 5 (CPZ+naringin+MCC950): Mice received CPZ for 6 weeks, after which naringin and MCC950 were co-administrated for 14 days, as mentioned above.

### 2.4 Y-maze Test

In this study, mice were subjected to a Y maze test to evaluate working memory before CPZ administration (Day 0), on the 42^nd^ day after CPZ consumption, and at the end of the relevant interventions (Day 56). The Y maze test was employed to evaluate the working memory [23]. The behavioral test was conducted in a quiet room with a controlled temperature of 23°C to ensure that the animals were not stressed during the test. Before introducing each animal to the testing area, the object was thoroughly cleaned with alcohol to prevent any distraction caused by the smell of animal waste.

The maze used in this study was constructed from wood and consisted of three arms (A, B, and C). During an eight-minute testing session, the mice were placed in the Y-maze and allowed to move in the maze freely. The number of arm entries was visually recorded. If the mouse explored all three arms of the maze in consequent explorations, the correct alternation was achieved. Alternatively, the mouse's failure to explore all three arms during three consecutive exploration instances was deemed an incorrect alternation. The percentage of each mouse's correct alternations was calculated by dividing the number of correct rotations by the total number of rotations and multiplying the result by 100 (number of correct alternations/(total number of input arms -2))*100 [24].

### 2.5 Samples Collection

Following the behavioral assessment, a mixture of ketamine (100 mg/kg) and xylazine (10 mg/kg) was administered to anesthetize the mice and sacrifice them. Subsequently, the brains were swiftly extracted, and two hemispheres were then separated. The right hemisphere was submerged in paraformaldehyde (PFA) for 12 h and then transferred to 30% sucrose solution at 4°C for immunohistofluorescence (IHF) and FluoroMyelin staining. The left hemispheres were stored at -70°C for gene expression analysis and biochemical assays.

### 2.6 Biochemical Assays

A homogenization step is required for evaluating the oxidant and antioxidant enzyme activity in a tissue sample. Typically, 10 to 20 mg of the corpus callosum of the left cerebral hemisphere of each animal was homogenized in PBS solution. The resultant lysate was centrifuged at 12,000 rpm for 15 minutes. The resulting supernatant, containing the target enzymes, was carefully isolated and transferred to a new tube. The tube was frozen at -70°C until further analysis.

#### 2.6.1 Malondialdehyde Assessment

Malondialdehyde (MDA) is a factor that results from lipid peroxidation and direct damage of oxidants to cellular polyunsaturated fatty acids (PUFA). To measure the concentration of MDA in a tissue homogenate, 0.5 mL homogenate was mixed with 1.5 mL 10% trichloroacetic acid (TCA) and centrifuged for 10 minutes. The resulting supernatant (2 mL) was mixed with 1 mL of 0.67% 2-thiobarbituric acid and heated in a boiling water bath for 30 min. Next, 2 mL of n-butanol solution was added to the solution and centrifuged. This experiment indicated a complex form of MDA with thiobarbituric acid, resulting in a pink-colored supernatant solution with an absorbance peak at a wavelength of 532 nm. The concentration of MDA was determined using 1,3,3-tetra-ethoxypropane as an external standard. The concentration of MDA was reported in units of nanomoles per milliliter (nmol/mL).

#### 2.6.2 Total Oxidant Status (TOS)

Assessment of the total oxidant status (TOS) level was based on Kiazist kit instructions. In brief, ferrous (Fe^+2^) ions, which are oxidized to ferric (Fe^+3^) ions in the presence of oxidants, produce a color in the presence of a chromogen. The color produced by the reaction had an absorbance peak within the range of 550-580 nm. The standard curve for the assay has been based on the relationship between the amount of oxidant present and the level of absorbance, with the standard curve being drawn in the presence of H_2_O_2_. The unit of measurement for the assay was in nanomoles per milliliter (nmol/mL).

#### 2.6.3 Total Antioxidant Capacity (TAC)

The total antioxidant capacity (TAC) refers to the overall amount of antioxidant substances present in a biological sample. The Kiazist kit utilizes cupric (Cu^+2^) ions, which results in a reduction of cupro (Cu^+1^) ions with present antioxidants, producing a color with the presence of chromogen. The color produced by the reaction has an absorbance peak at a wavelength of 450 nm. The TAC value of the sample was expressed in units of nanomoles of trolox equivalent per milliliter (nmol of trolox equivalent/mL).

#### 2.6.4 Superoxide Dismutase (SOD)

Superoxide dismutase (SOD) prevents oxidative stress by neutralizing the superoxide ion (O_2_^−^). Its enzyme activity can be measured with high sensitivity calorimetrically using a Kiazist kit. The assay utilizes an absorbance peak at a wavelength of 570 nm. The enzyme activity was reported as the percentage of inhibition (inhibition rate %), reflecting the degree to which the enzyme can inhibit the oxidation of a substrate.

#### 2.6.5 Catalase Activity

Catalase (CAT) is an enzyme that neutralizes hydrogen peroxide. The Kiazist Kit utilizes the peroxidase activity of catalase in the presence of methanol, which is then stopped by the addition of its restrainer. The formaldehyde produced as a result of the reaction between catalase and methanol would react with purpald, to produce a purple color. This dye has an absorbance peak at a wavelength of 540 nm. The enzyme activity was reported in units of nanomoles of the substrate (hydrogen peroxide) consumed per minute per milliliter (nmol/min/mL) or milliunits per milliliter (mU/mL).

#### 2.6.6 Glutathione Peroxidase Activity

An essential antioxidant enzyme that prevents oxidative stress is glutathione peroxidase (GPx). It functions by converting hydrogen peroxide to water and using glutathione in the process. The Kiazist kit employs a reaction method known as coupling, which involves the use of the enzyme glutathione reductase and its coenzyme NADPH. The enzyme activity was reported in units of nanomoles of glutathione (GSH) oxidized per minute per milliliter (nmol of GSH oxidized/min/mL).

### 2.7 Quantitative Real-time PCR (qRT-PCR) Analysis

Brain homogenates were utilized by a TRIzol reagent (Invitrogen) to extract total RNA. The RNA samples' purity and quantity were evaluated using a spectrophotometer (Nanodrop 2000, Thermo Fisher), demonstrating an optical density (OD) 260/280 nm ratio ranging between 1.8 and 2.0 for all samples. Identical amounts of RNA (300 ng) were then reverse-transcribed to cDNA according to the manufacturer's instructions using a ParsTous kit. The qRT-PCR analysis was performed using SYBR^®^ Green PCR Master Mix (Ampliqon, Herlev, Denmark, Cat. NO. 250507) and specific primers for *Nrf2*, *GSK3β*, *HO1*, *NQO-1*, *IL1β*, *IL-18*, *MBP*, *PDGFRα*, *Olig2*, and *HPRT* (Pishgam Biotech, Iran), as reported in Table **1**. The cycling PCR reaction was carried out as follows: The thermal cycling protocol involved an initial denaturation step at 95°C for 15 minutes, succeeded by 35 cycles consisting of denaturation at 95°C for 20 seconds and annealing/extension at 60°C for 60 seconds. Gene expression levels were determined utilizing the 2^^-ΔΔCt^ formula. *HPRT* mRNA levels were employed as the internal control for normalization purposes.

### 2.8 Histological Examinations

#### 2.8.1 FluoroMyelin Staining

FluoroMyelin staining was conducted to assess the extent of demyelination in accordance with the manufacturer's instructions for FluoroMyelin™ Green Fluorescent Myelin Stain (F34651, Invitrogen, MA, USA). Frozen tissue sections of the corpus callosum were under three washes with PBS and then incubated with FluoroMyelin solution at room temperature for 20 minutes. Subsequently, the sections were washed with PBS and labeled with a 4′,6-diamidino-2-phenylindole (DAPI) solution at an appropriate dilution. Visualization of the sections was performed using a fluorescence microscope (Olympus, Japan) [25]. Image J software (version 1.42 V, NIH, USA) was employed to calculate the demyelinated area percentage to measure demyelination levels.

#### 2.8.2 Immunohistofluorescence

The right brain samples were embedded in optimal critical temperature (OCT), where a cryostat (Histo-Line Laboratories, Milan, Italy) was used for sample-sectioning. Coronal sections with a thickness of 6 μm were taken from the region of the corpus callosum. The sections were placed on charged slides at room temperature. After being PBS-washed, the tissue sections were incubated with Triton X100 0.3% for 20 minutes. Then, normal goat serum (NGS) of 10% was used to block non-specific bindings. Subsequently, the slices were incubated overnight at 4°C with primary antibodies against GFAP (1/400) as an astrocyte marker and MOG (1/100) as a myelin marker. After PBS-washing the tissue sections three times, the appropriate secondary antibodies (1/1000) were added for 1 hour at room temperature. Following another three washes, DAPI was added for nuclear staining. Subsequently, the stained slides underwent evaluation by a fluorescence microscope (Olympus, Japan). The quantification of immunostaining data was carried out using Image J software (version 1.42 V, NIH, USA) in a blinded manner. For histological analysis, three sections from each specimen, three specimens from each animal, and three mice in each group were evaluated blindly. Regarding astrocyte counting, the average number of GFAP-positive cells per field was calculated. For MOG quantification, images were assessed following a previously described technique [26].

### 2.9 Statistical Analysis

The statistical analysis was performed using GraphPad Prism 9.0 (GraphPad Software Inc., San Diego, CA, USA). The data are expressed as mean ± SEM. The Y maze data were analyzed using repeated measures and a Bonferroni post-test. The biochemical results and gene expression data were evaluated using one-way ANOVA followed by Tukey post hoc. The histological data were analyzed using a non-parametric Kruskal Wallis test and Dunn’s post-test. Statistical significance was defined as *P <* 0.05.

## 3 RESULTS

### 3.1 Co-administration of Naringin and MCC950 Improved the Working Memory in CPZ-receiving Mice

**Fig. (1):**
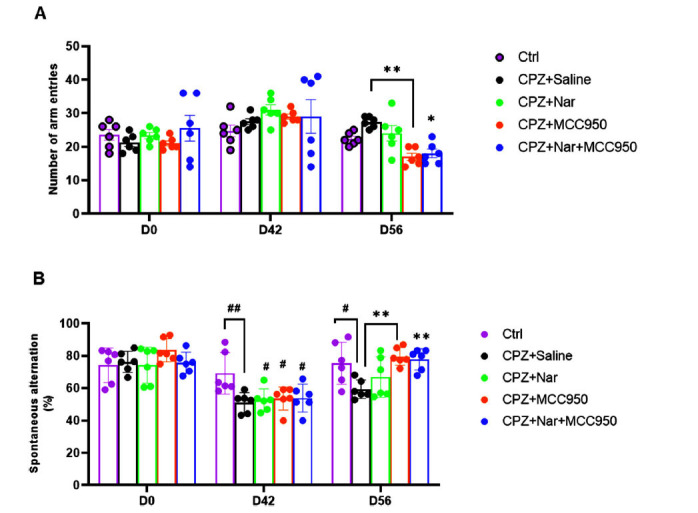
The effect of MCC950 and naringin administrations on mice exploratory behavior in demyelination model induced by CPZ. (**A**) The number of arm entries in the Y-maze test. *P < 0.05 and **P < 0.01 compared to the CPZ+Saline. (**B**) Spontaneous alternation. ^#^*P* <0.05 and ^##^*P <* 0.01 compared to the Ctrl (n=6 per group).

Y-maze data analysis revealed a significant decline in the arm entries number in CPZ+MCC950 and CPZ+Nar+ MCC950-treated mice compared to CPZ+Saline on day 56 (*P* = 0.005 and *P* = 0.01, respectively). The number of arm entries was not significantly different before receiving relevant interventions on days 0 or 42 (Interaction F (8, 75) = 2.53, *P* = 0.016; Treatment F (4, 75) = 1.63, *P* = 0.17; Time F (2, 75) = 14.74, *P <* 0.0001; Fig. **1A**).

There was no significant difference in spontaneous alternation between experimental groups on day 0. However, after 42 days, the percentage of spontaneous alternation was notably reduced in all CPZ-treated groups compared to the Control group (*P <* 0.05). By day 56, the percentage of spontaneous alternation increased in the CPZ+MCC950 and CPZ+Nar+MCC950 groups compared to the CPZ+Saline group (*P <* 0.01). Additionally, there was a significant difference in spontaneous alternation observed between the Control and CPZ+Saline groups on day 56 (*P* = 0.02) (Interaction F (8, 75) = 2.89, *P* = 0.007; Treatment F (4, 75) = 5.05, *P* = 0.001; Time F (2, 75) = 44.44, *P <* 0.0001; Fig. **1B**).

### 3.2 Co-administration of Naringin and MCC950 Increased the Gene Expression of Oligodendrocyte Lineage Cells

**Fig. (2):**
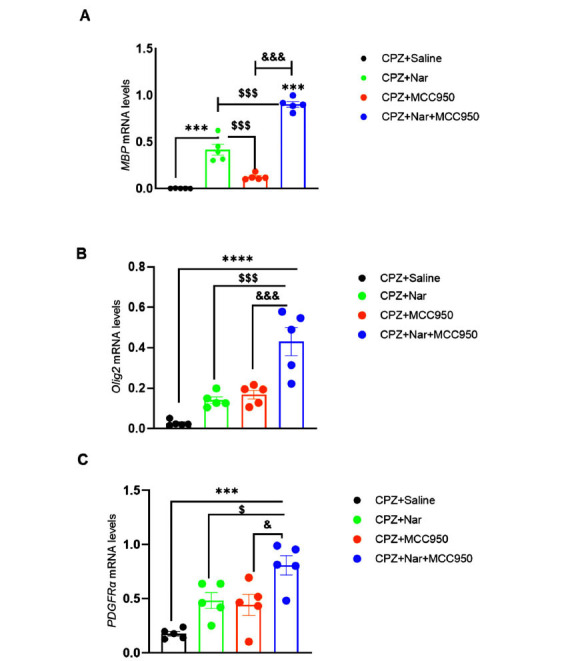
The impact of naringin and MCC950 on *MBP* (**A**), *Olig2* (**B**), and *PDGFRα* (**C**) gene expression in the demyelination model induced by CPZ. The MBP, Olig2, and PDGFRα expression levels were evaluated relative to HPRT, the housekeeping gene. Data are presented as mean ± SEM. Statistical significance is denoted as ***P < 0.001 and ****P < 0.0001 compared to CPZ+Saline; ^$^*P <* 0.05 and ^$$$^*P <* 0.001 compared to CPZ+Nar; and ^&^*P <* 0.05 and ^&&&^*P <* 0.001 compared to CPZ+MCC950 (n = 5 per group).

According to Fig. (**2A**), the *MBP* gene expression was only significant in CPZ+Nar and CPZ+Nar+MCC950 in comparison with the CPZ+Saline group (F (3, 16) =142.6; *P <* 0.001). Further, a significant increase was observed in the expression of *MBP* in CPZ+Nar+MCC950 treated mice compared to the CPZ+MCC950 and CPZ+Nar groups (*P <* 0.001). Furthermore, the results indicated that the administration of CPZ+Nar had a greater impact on MBP gene expression compared to the CPZ+MCC950 groups (*P <* 0.001) (refer to Fig. **2A**).

In addition, *Olig2* expression substantially increased in CPZ+Nar+MCC950 compared with CPZ+Saline (F (3, 16) =20.99; *P <* 0.001) (Fig. **2B**). The difference in *Olig2* expression between CPZ+Nar+MCC950 receiving mice and CPZ+Nar and CPZ+MCC950 groups (*P =* 0.0003 and *P =* 0.0007, respectively) was also significant (Fig. **2B**).

Gene expression analysis indicated that the levels of *PDGFRα* rose in the CPZ+Nar+MCC950 in comparison to the CPZ+Saline (F (3, 16) =11.72; *P =* 0.0003). In addition, PDGFRα gene expression showed a significant difference in CPZ+Nar+MCC950 compared to CPZ+Nar and CPZ+ MCC950 (*P =* 0.034 and *P =* 0.016, respectively), significantly. In spite of the increased *PDGFRα* gene expression in CPZ+Nar and CPZ+MCC950 treated mice, this growth was not statistically significant compared to the CPZ+Saline group (Fig. **2C**).

### 3.3 Co-administration of Naringin and MCC950 Improved Myelination of the Corpus Callosum

**Fig. (3):**
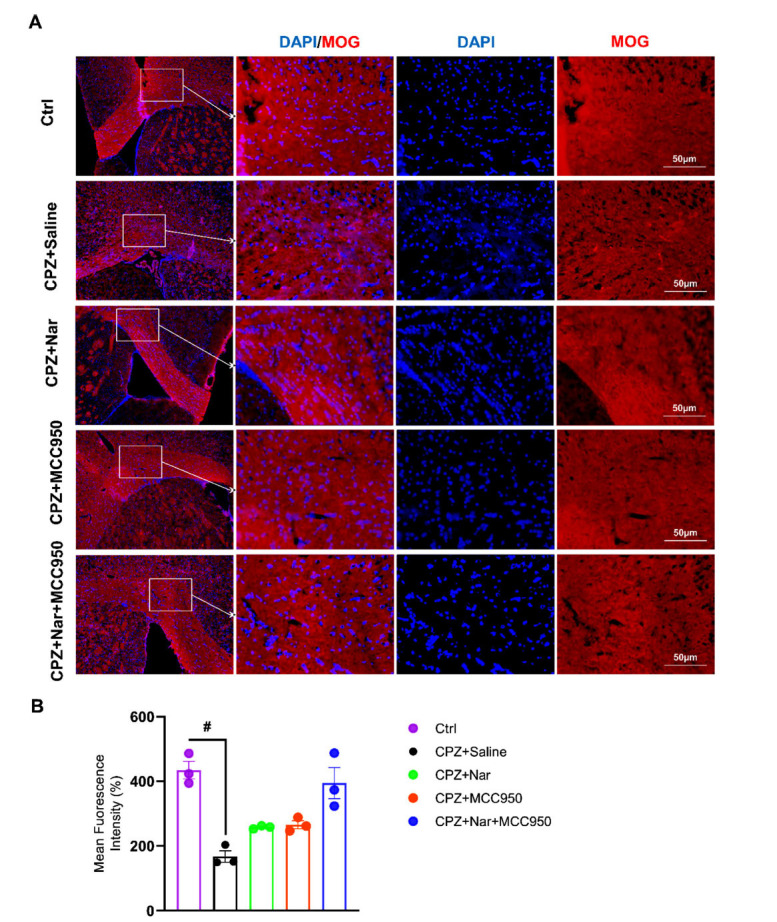
Co-administration of naringin and MCC950 increased the immunointensity of MOG in the corpus callosum. (**A**) Immunostaining against MOG (red) as a marker myelinating OLs in the corpus callosum, with DAPI (blue) that labels nuclei (Scale bar: 50 μm). (**B**) Immunostaining quantitative analysis against MOG revealed ^#^*P =* 0.026 compared to the Ctrl group (n = 3 per group).

Analysis of MOG intensity revealed that its immunointensity was significantly lower in the CPZ+Saline compared to the Ctrl group (P = 0.026) (X2= 56.42, df =8, *P <* 0.0001) (Figs. **3A**, **B**).

### 3.4 Co-administration of Naringin and MCC950 Reduced the Extent of Demyelination areas in the Corpus Callosum

**Fig. (4):**
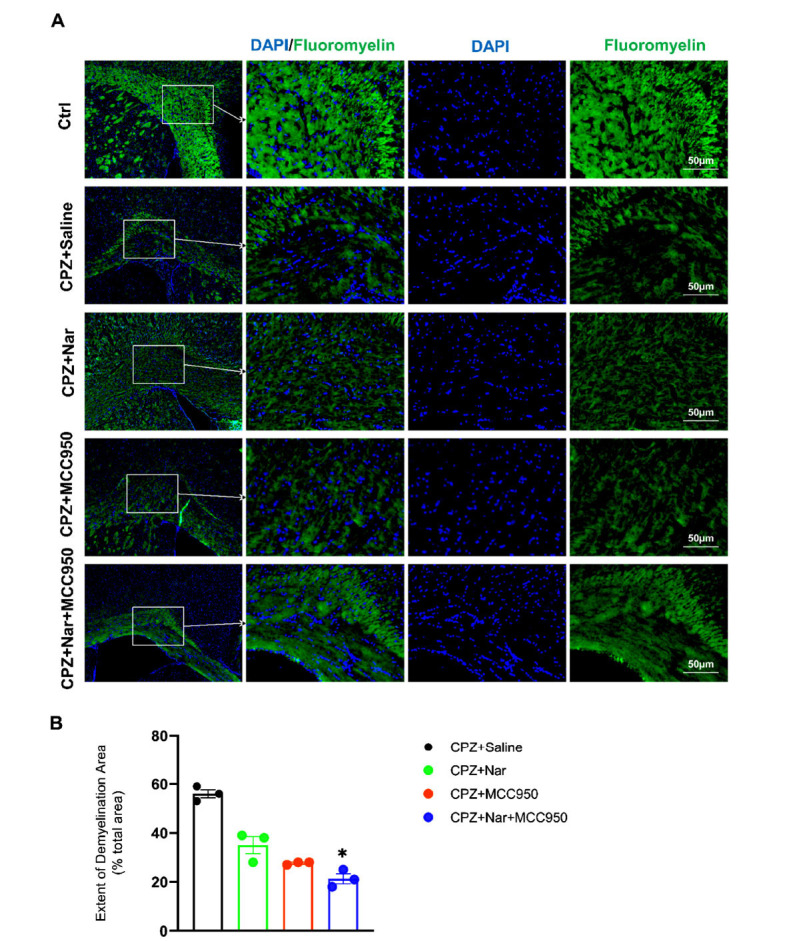
Co-administration of naringin and MCC950 alleviated the demyelination areas extent in the corpus callosum. (**A**) FluoroMyelin staining (green) in the corpus callosum, DAPI (blue) used for nuclei labeling (Scale bar: 50 μm). (**B**) Quantitative analysis of FluoroMyelin staining revealed ^*^P = 0.012 compared to the CPZ+Saline group (n = 3 per group).

To examine the effect of naringin and MCC950 administration on demyelination level, the extent of the demyelination area was assessed by Fluoromyelin staining on day 14 after treatment. The results exhibited that the demyelination areas extent diminished in the CPZ+Nar+MCC950 treated mice compared to the CPZ+Saline group (*P =* 0.012) (X2=2.34, df=6, *P =* 0.88) (Fig. **4A**, **B**).

### 3.5 Co-administration of Naringin and MCC950 Improved the Antioxidant Enzyme Activities in the Corpus Callosum

According to the data presented in Fig. (**5A**), administration of naringin and MCC950 resulted in a modest increase in SOD activity, albeit showing an insignificant difference. Conversely, CPZ treatment decreased SOD activity compared to the control group. However, the naringin and MCC950 co-administration mitigated the negative impact of CPZ and resulted in a significant increase in SOD activity (F (4, 15) =3.49; *P =* 0.033).

**Fig. (5):**
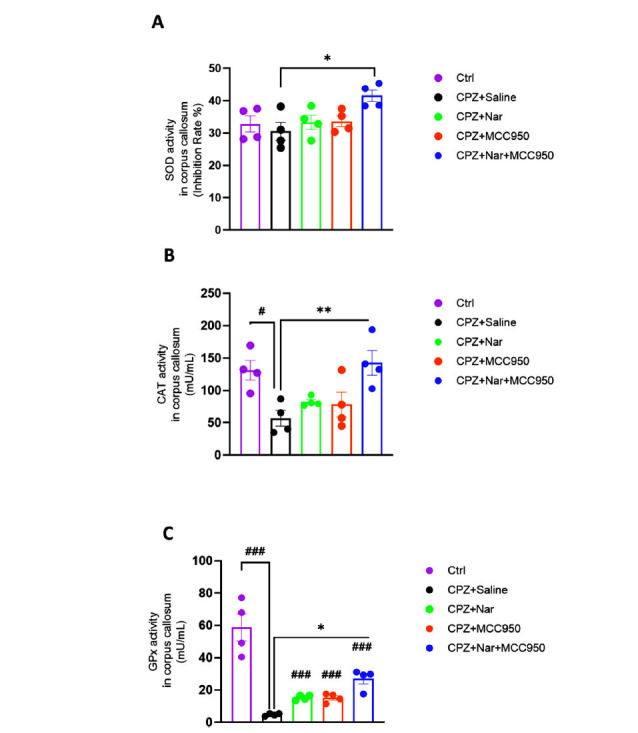
The effect of naringin and MCC950 on SOD A), CAT B), and GPx C) levels in corpus callosum. ^#^*P <* 0.05 and ^###^*P <* 0.001 compared to the Ctrl; *P < 0.05, **P < 0.01 and ***P < 0.001 compared to the CPZ+Saline (n=4 per group).

Administration of CPZ led to a significant decline in CAT activity compared to control mice (*P =* 0.022). Interestingly, co-administration of naringin and MCC950 significantly boosted the CAT activity (F (4, 15) =6.08; *P =* 0.004) (Fig. **5B**).

As depicted in Fig. (**5C**), CPZ+Saline, CPZ+Nar, CPZ+MCC950, and CPZ+Nar+MCC950 experimental groups GPx activity was reduced in comparison with the control group (*P <* 0.001) notably. However, the application of CPZ+Nar+MCC950 led to a significant increase in tissue GPx activity in comparison with the CPZ+Saline group (F (4, 15) =26.08; *P <* 0.001).

### 3.6 Co-administration of Naringin and MCC950 Improved the Antioxidant Capacity and Lipid Peroxidation in the Brain Tissue

**Fig. (6):**
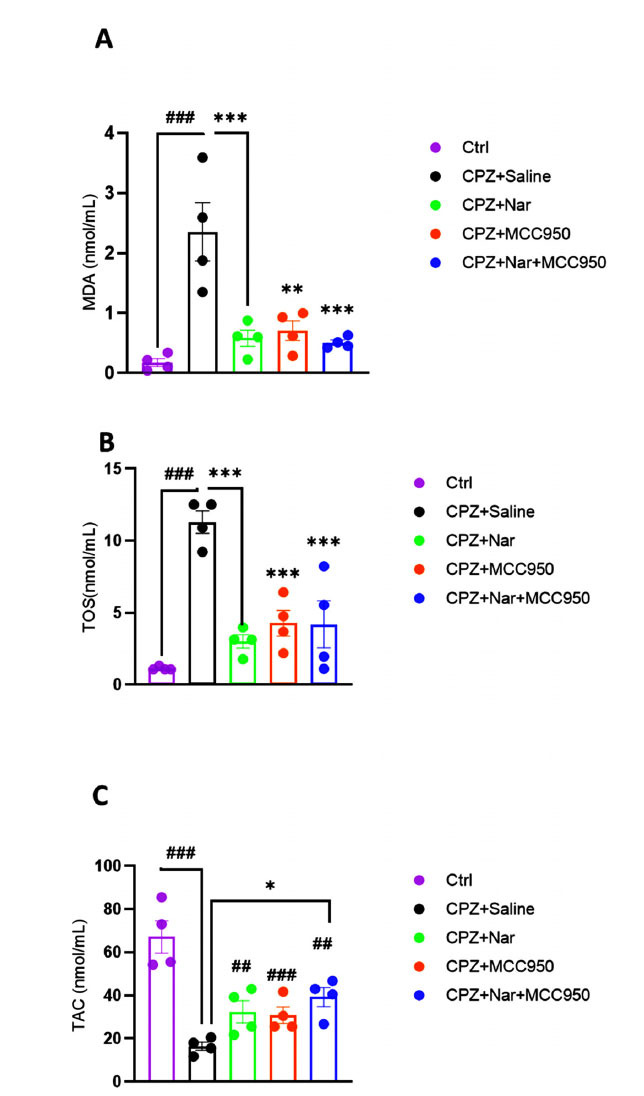
The effects of naringin and MCC950 on MDA (**A**), TOS (**B**), and TAC (**C**) in the demyelination model induced by CPZ. A) ^##^*P <* 0.01 and ^###^*P <* 0.001 compared to the Ctrl. *P < 0.05, **P < 0.01 and *** *P <* 0.001 compared to the CPZ+Saline (n=4 per group).

The MDA levels were significantly elevated in the CPZ+Saline group compared to the Ctrl group (*P <* 0.001). In contrast, administration of naringin, MCC950, or Nar+MCC950 significantly reduced the CPZ-induced elevation of MDA ((F (4, 15) =12.80; *P <* 0.001) (Fig. **6A**).

Administration of CPZ significantly augmented TOS levels in comparison with the control group (*P <* 0.001). However, TOS production was significantly reduced in CPZ+Nar, CPZ+MCC950, or CPZ+Nar+MCC950 treated mice in comparison with CPZ+Saline (F (4, 15) =17.10; *P <* 0.001) (Fig. **6B**).

In addition, a notable reduction in TAC levels was observed in all experimental groups in comparison to the control group (*P <* 0.001 in CPZ+Saline, *P =* 0.001 in CPZ+Nar, *P <* 0.001 in CPZ+MCC950, and *P =* 0.008 in CPZ+Nar+MCC950). The level of TAC was significantly heightened in CPZ+Nar+MCC950 receiving mice in comparison to the CPZ+Saline (*P =* 0.034) (F (4, 15) =14.50; *P <* 0.001) (Fig. **6C**).

### 3.7 Co-administration of Naringin and MCC950 Boosted the Antioxidant’s Genes Expression Levels

**Fig. (7):**
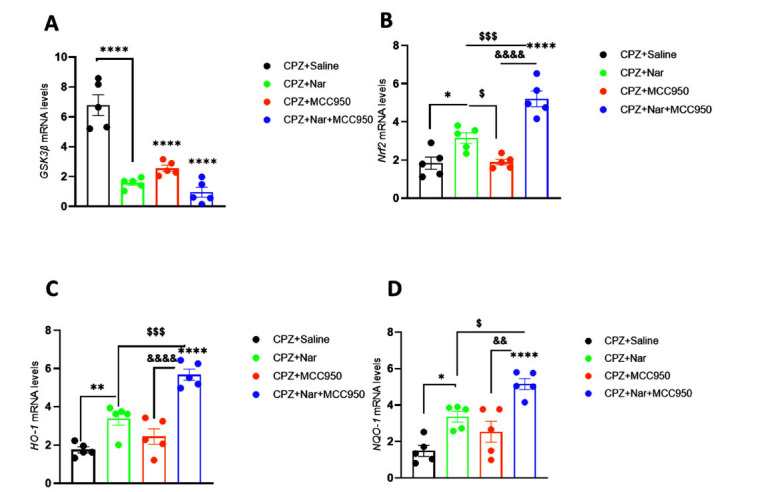
The effect of Naringin and MCC950 on *GSK-3β* (**A**), *Nrf2* (**B**), *HO-1* (**C**), and *NQO-1* (**D**) genes expression in demyelination model induced by CPZ. *GSK-3β*, *Nrf2*, *HO-1*, and *NQO-1* were measured in relation to *HPRT* as an internal control gene. Data expression is mean ± SEM. *P < 0.05, **P < 0.01 and ****P < 0.0001 *vs.* CPZ+Saline; ^$^*P <* 0.05, ^$$$^*P <* 0.001 and ^$$$^*P <* 0.0005 *vs.* CPZ+Nar; ^&&^*P <* 0.01 and ^&&&&^*P =* 0.0001 *vs.* CPZ+MCC950 (n=5 per group).

The RT-qPCR analysis demonstrated a significant reduction in the relative mRNA expression level of *GSK3β* in the corpus callosum following administration of CPZ+Nar, CPZ+MCC950, or CPZ+Nar+MCC950 compared to the CPZ+Saline group (F (3, 16) =41.29; *P <* 0.0001) (Fig. **7A**).

The application of CPZ+Nar+MCC950 and CPZ+Nar led to a significant increase in the relative mRNA expression levels of the *Nrf2* gene in the corpus callosum compared to the CPZ+Saline group ((F (3, 16) =27.35; *P <* 0.0001, *P =* 0.03, respectively). The expression of *Nrf2* was enhanced in CPZ+Nar+MCC950 and CPZ+MCC950 groups compared to the CPZ+Nar treated mice significantly (*P =* 0.001 and *P =* 0.04, respectively). Furthermore, a higher expression level of *Nrf2* was observed in CPZ+Nar+ MCC950 receiving mice in comparison with the CPZ+ MCC950 (*P <* 0.0001) (Fig. **7B**).

CPZ+Nar or CPZ+Nar+MCC950 administration resulted in a notable elevation in the relative mRNA expression levels of the *HO-1* gene in comparison with the CPZ+Saline group ((F (3, 16) =29.80; *P <* 0.0001). The *HO-1* expression was also significantly different between CPZ+Nar+MCC950 and CPZ+Nar treated mice (*P =* 0.0005). Interestingly, co-administration of naringin and MCC950 had a stronger effect on *HO-1* gene expression compared to the CPZ+MCC950 group (*P <* 0.0001) (Fig. **7C**).

A significant increase in *NQO-1* levels was found in CPZ+Nar and CPZ+Nar+MCC950 treated mice compared to the CPZ+Saline group ((F (3, 16) =16.21; *P <* 0.0001). There was also a significant difference between CPZ+Nar+ MCC950 and CPZ+Nar treated mice (*P =* 0.02). In addition, co-administration of naringin and MCC950 had a greater effect on the expression of NQO-1 than that of the CPZ+ MCC950 group (*P =* 0.001) (Fig. **7D**).

### 3.8 Co-administration of Naringin and MCC950 Alleviated the Inflammation Level

**Fig. (8):**
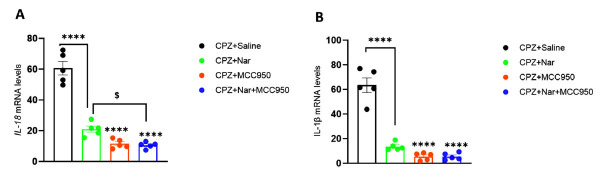
Impact of naringin and MCC950 on *IL-18* (**A**) and *IL-1β* (**B**) genes expression in demyelination model induced by CPZ. Data expression is mean ± SEM. ****P <0.0001 compared to the CPZ+Saline and ^$^*P =* 0.046 compared to the CPZ+Nar (n=5 per group).

The expression levels of *IL-18* and *IL-1β* were notably reduced in CPZ+Nar, CPZ+MCC950, or CPZ+Nar+ MCC950 treated mice compared to the CPZ+Saline group ((F (3, 16) =86.67, F (3, 16) =77.63; *P* < 0.0001, respectively). Furthermore, the CPZ+Nar+MCC950 group exhibited a significant reduction in *IL-18* expression compared to the CPZ+Nar treated mice (*P* < 0.04) (Fig. **8A**, **B**).

### 3.9 Co-administration of Naringin and MCC950 Ameliorated Astrocyte Activation in the Corpus Callosum

**Fig. (9):**
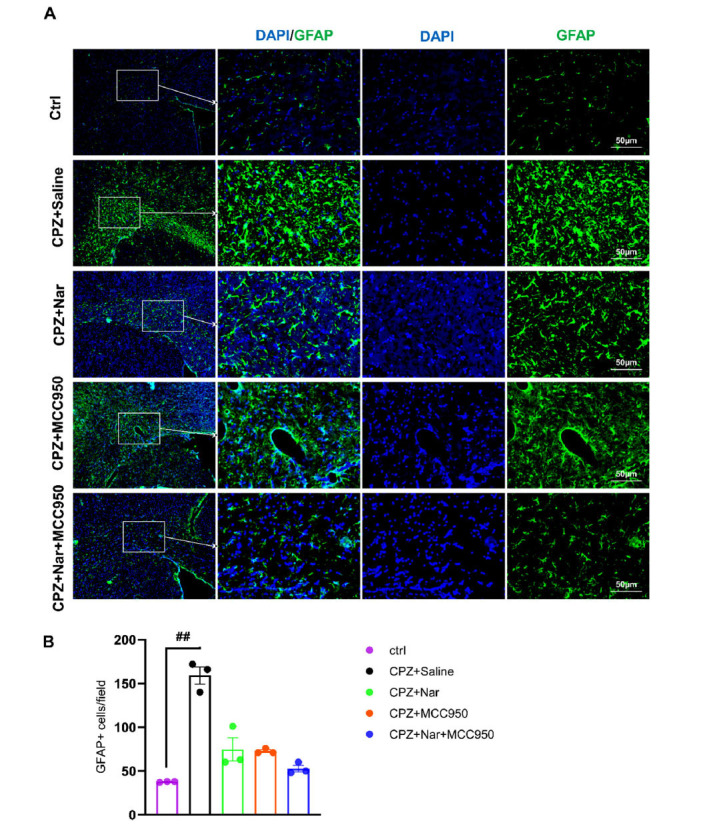
Administration of naringin and MCC950 ameliorated the number of activated astrocytes in the corpus callosum of mice. (**A**) Immunostaining against GFAP (green), pointing astrocytes in the corpus callosum. DAPI (blue) labels nuclei (Scale bar: 50 μm). (**B**) immunostaining quantification data against GFAP. ^##^*P* = 0.01 compared to the Ctrl group (n=3 per group).

To evaluate the level of astrocyte activation, GFAP immunostaining was performed on brain sections. The number of GFAP-positive cells was significantly higher in the CPZ+Saline group compared to the Ctrl group (*P* = 0.01) (X2= 18.51, df =8, *P =* 0.017) (Figs. **9A**, **B**). There was no significant difference between the experimental groups.

## 4 DISCUSSION

Different pieces of evidence suggest that MCC950, as an inhibitor of NLRP3, attenuates the inflammatory response. In addition, naringin, as a flavonoid compound, has presented remarkable antioxidant properties [27, 28].

Here, we investigated the probable synergistic effect of naringin alongside MCC950 on various CPZ-induced demyelination aspects, including working memory, extent of demyelination, myelin repair, oxidative stress, astrocyte activation, and inflammation. Our results indicated the effective enhancement of working memory, reduction of demyelinating areas, and myelin repair promotion through the naringin and MCC950 combination. Additionally, we observed a reduction in oxidative stress, astrocyte activation, and inflammation in the corpus callosum of naringin and MCC950-treated mice.

Firstly, we found that administration of MCC950 or naringin+MCC950 would improve the working memory. The positive impact of MCC950 or naringin+MCC950 on working memory was attributed to their influence on locomotion activity and the modulation of initial spontaneous alternation behavior. Consistent with our data, previous reports suggested that CPZ causes cognitive decline and myelin degradation [29]. Hongyu *et al*. demonstrated that CPZ results in white matter damage and disturbances of behavioral aspects in the Y-maze test [30]. Furthermore, Meng *et al*. reported that naringin could improve cognitive functions by affecting oxidative stress, cell apoptosis, and the cholinergic system in the hydrocortisone-induced memory deficit model [31]. In a dementia mouse model, Ren *et al*. revealed that MCC950 treatment alleviates cognitive dysfunction, suppresses the NLRP3 expression, and reduces the hippocampal astrocytes as well as microglia activation [32].

We could not find any significant difference in Nar+MCC950 treated mice compared to the MCC950 or naringin alone. The dosage and duration of treatment naringin might not be sufficient to observe combined effects in behavioral tests. It has been shown that administering naringin *via* gavage leads to a reduced anti-inflammatory effect compared to its intraperitoneal (i.p.) injection. Prolonging the screening period may potentially boost the efficacy of naringin in improving memory function.

In the second phase, the effect of naringin and MCC950 administration was examined on the demyelination level of the corpus callosum. Myelin staining revealed that the extent of demyelination areas diminished in MCC950 or naringin+MCC950-treated mice.

Shao *et al*. discovered that MCC950 treatment reduced microglial activation and CPZ-induced myelin damage in mice [3], corroborating our findings. Additionally, Hou *et al*. illustrated that MCC950 mitigated neuronal damage, demyelination, and decreased levels of oligodendrocytes in EAE mice by inhibiting the NLRP3 inflammasome [33]. Feng *et al*. observed that naringin possessed the capability to cross the BBB, thereby safeguarding brain tissue and modulating brain chemistry [34]. Furthermore, Rong *et al*. reported that naringin enhanced the myelin sheath density and size, as well as the myelinated nerve fibers quality through oligodendrocyte precursor cells stimulation and promoting the remyelination process in the spinal cord injury model [13].

In the third phase of our study, our data demonstrated that the remyelination process was enhanced following the co-administration of naringin and MCC950. To investigate this co-administration impact on myelination, we evaluated the *MBP*, *Olig2*, and *PDGFRα* genes expression, along with MOG immunostaining in the corpus callosum of mice. A significant increase was found in gene expression of *MBP*, *Olig2*, and *PDGFRα,* as well as MOG immunointensity following administration of naringin and MCC950.

Stidworthy *et al*. also showed the demyelination presence and depletion of OLs within the initial three weeks of CPZ administration was succeeded by a process of remyelination after a period of 5 to 6 weeks [35]. *MBP,* in various investigations, has been shown to play a role as a constituent of the myelin sheath [36]. Abd El Aziz *et al*. demonstrated that demyelination with reduced MBP protein and mRNA expression occurred in CPZ-fed mice [1]. According to reports, intragastric administration of naringin resulted in enhanced remyelination in rats with spinal cord injury. This study found that naringin significantly attenuated white matter demyelination and improved the myelinated axon quality by elevating *MBP* levels [13].

According to another study by Rong *et al*., naringin increased the *Olig2* protein level by reducing OPC death and enhancing their survival in the spinal cord injury model [13]. Wegener *et al*. found that *Olig2* expression promotes the OPCs migration and differentiation into mature OLs, which leads to increased remyelination [37]. Furthermore, prior studies demonstrated that *PDGF* functions as a survival factor in OL maturation [38]. Previous data also showed that activation of *PDGF-α* ligand would induce OPC proliferation by stimulating *PDGFRα* signaling in acute experimental demyelination [39].

To explore the impact of naringin and MCC950 on oxidative stress levels, we assessed the antioxidant markers activity and expression of the genes related to oxidative stress—*Nrf2*, *HO-1*, *NQO1*, and *GSK3β*—in the mice corpus callosum. Our results unveiled that CPZ administration led to a decline in *Nrf2* expression, as well as a reduction in levels of *HO-1* and *NQO-1*, accompanied by an increase in *GSK3β* levels. These findings underscore the pivotal role of this signaling pathway in demyelination induced by CPZ. In a parallel study, the inhibitory effect of CPZ was evaluated on the *Nrf2*, *HO-1*, and *NQO-1* expression in the corpus callosum of mice [1]. Additionally, Draheim *et al*. demonstrated that activation of *Nrf-2* mitigated the loss of OLs and demyelination in CPZ-induced demyelination [40]. Furthermore, a recent study has shown that *GSK-3β* suppresses *Nrf2* [41]. Xing *et al*. demonstrated that the *GSK-3β* depletion preserved myelin thickness and reduced glial activation. This protection would also prevent OLs from undergoing apoptosis and demyelination reduction in the acute CPZ model [42].

Additionally, it was indicated that GPX, CAT, and SOD activities were reduced after 6 weeks of CPZ consumption, leading to a diminished capacity to neutralize ROS. Furthermore, exposure to CPZ results in a reduction in TAC levels and an increase in MDA and TOS levels. Consistent with our results, previous evidence indicated that CPZ administration reduced the cellular antioxidant levels, particularly GSH, and elevated the brain MDA levels [43].

The combined administration of naringin and MCC950 successfully mitigated the CPZ-induced elevation in TOS and MDA levels, concurrently boosting the GPx, CAT, SOD, and TAC levels. Consistent with these findings, significant upregulation was observed in the *Nrf2*, *HO-1*, and *NQO1* gene expression, with a reduction in *GSK3β* levels in mice treated with naringin and MCC950. These results suggested that co-administration of naringin and MCC950 activates the Nrf2 pathway, which protects CPZ-induced oxidative stress.

Similar to our study, several studies have indicated that the most effective stimulators of *Nrf2* are flavonoids, leading to the expression of genes responsible for cellular protection. The highest level of *Nrf2* was found in naringin-treated animals [44]. Naringin lowered the ROS levels, possibly through direct scavenging ROS or by increasing the antioxidant gene expression [16]. Furthermore, Shi *et al*. suggested that MCC950 administration enhanced the *Nrf2* expression in isoproterenol-induced oxidative stress. This finding suggests a possible link between MCC950 and *Nrf2* [45]. Ni *et al*. showed that MCC950 could stimulate the Nrf2/HO-1/ NQO1 pathway and alleviate oxidative stress in mouse chondrocytes [46].

Additionally, our data revealed that co-administration of naringin and MCC950 reduced the level of astrocyte activation. Based on numerous studies, CPZ-induced gliosis is characterized by the excessive proliferation of microglia and astrocytes [47]. Gopinath *et al*. showed that GFAP, a widely acknowledged marker of gliosis, is overexpressed in neuroinflammatory diseases [48]. Consistent with our study, further results revealed a GFAP expression reduction by naringin, leading to astroglial cell inactivation and, hence, reduced production of inflammatory mediators and reactive gliosis associated with 3-NP-induced neurodegeneration [48].

Consistent with our study, another investigation revealed that MCC950 indirectly inhibited astrocytes by inhibiting microglia, as activated neuroinflammatory microglia stimulate astrocytes [49]. In contrast, a previous report has found that MCC950 did not show effectiveness in reducing the activation of astrocytes [50]. The observed discrepancy can be due to different sampling times in the two experiments, which were conducted in different phases, peak phase, and late phase. Our hypothesis was that transient suppression of the NLRP3 inflammasome by MCC950 might reduce the activation of astrocytes.

In our investigation, CPZ significantly enhanced the expression levels of *IL-1β* and *IL-18*. Conversely, the administration of naringin, MCC950, or their combination effectively reduced the expression of these pro-inflammatory cytokines in the corpus callosum of mice. As mentioned earlier, microgliosis became apparent within a week of CPZ administration and continued to increase in CPZ exposure from the third to fourth week [51]. According to evidence, activation of microglia can lead to the generation of pro-inflammatory cytokines, which may have detrimental effects on neurons and other glial cells [52]. It has been shown that MCC950 effectively suppresses the NLRP3 inflammasome activity and reduces the secretion of pro-inflammatory cytokines such as *TNF-α*, *IL-1β*, and *IL-18* in the spinal cord injury model [28]. Vafeiadou *et al*. demonstrated that naringin, as a potent flavonoid, effectively attenuated the astrocytes and microglia inflammatory response by inhibiting p38 and STAT-1, thereby mitigating neuronal damage [53].

## CONCLUSION

The findings of this study suggest that co-administration of naringin and MCC950 can exert a beneficial effect on CPZ-induced demyelination and working memory impairment in mice. The positive effects of naringin and MCC950 are, in part, attributed to their ability to reduce oxidative stress, astrocyte activation, and inflammation. Furthermore, our data indicate the involvement of Nrf2 signaling pathways in mediating the protective effects of naringin and MCC950.

## Figures and Tables

**Table 1:** The primer sequences used in the present study.

**Gene**	**Forward 5’→ 3’**	**Reverse 5’→ 3’**
*Nrf2*	CCAGCACATCCAGACAGACACC	GGCAAGCGACTCATGGTCATCTAC
*GSK3β*	GGCTGTGTGTTGGCTGAATTGTTG	TTGCTCCCTTGTTGGTGTTCCTAG
*HO-1*	TGAACACTCTGGAGATGACACCTG	CTGTGAGGGACTCTGGTCTTTGTG
*MBP*	CAGAGACACGGGCATCCTTGAC	GCAGGGAGCCATAATGGGTAGTTC
*NQO-1*	CAGCCAATCAGCGTTCGGTATTAC	AGCCTCTACAGCAGCCTCCTTCA
*Olig2*	CTTATTACAGACCGAGCCAACACC	GACGATGGGCGACTAGACACC
*PDGFR*α	AAGTGGAAGAGACCATCGCAGTTC	TGTGAGTTCAGATCGCAGAGTGG
*IL-18*	CGCCTCAAACCTTCCAAATCACTTC	CAAAGTTGTCTGATTCCAGGTCTCC
*IL1β*	ATGCCACCTTTTGACAGTGATGAG	ATGTGCTGCTGCGAGATTTGAAG
*HPRT*	ATTATGCCGAGGATTTGGA	ACTTATAGCCCCCCTTGA

## Data Availability

Not applicable.

## LIST OF ABBREVIATIONS

ASC: Apoptosis Associated Speck-like Protein Containing a CARD
BBB: Blood-brain Barrier
CARD: Caspase Recruitment Domain
CAT: Catalase
CNS: Central Nervous System
CPZ: Cuprizone
EAE: Experimental Autoimmune Encephalomyelitis
GFAP: Glial Fibrillary Acidic Protein
GPx: Glutathione Peroxidase
GSH: Glutathione
GSK-3β: Glycogen Synthase Kinase-3β
HO-1: Heme Oxygenase-1
HPRT: Hypoxanthine-guanine Phosphoribosyl Transferase
IL: Interleukin
MBP: Myelin Basic Protein
MDA: Malondialdehyde
MOG: Myelin Oligodendrocyte Glycoprotein
MS: Multiple Sclerosis
NADPH: Nicotinamide Adenine Dinucleotide Phosphate
NF-κB: Nuclear Factor Kappa B
NGS: Normal Goat Serum
NLR: NOD-like Receptor
NLRP3: NLR Family Pyrin Domain Containing 3
NQO-1: NAD(P)H Quinone Acceptor Oxidoreductase 1
Nrf2: Nuclear Factor Erythroid 2-related Factor
OCT: Optimal Critical Temperature
Olig2: Oligodendrocyte Transcription Factor 2
OLs: Oligodendrocytes
OPC: Oligodendrocyte Precursor Cells
PBS: Phosphate-buffered Saline
PDGFRα: Platelet-derived Growth Factor Receptor α
PUFA: Polyunsaturated Fatty Acids
qRT-PCR: Quantitative Real-time PCR
ROS: Reactive Oxygen Species
SOD: Superoxide Dismutase
TCA: Trichloroacetic Acid
TNF-α: Tumor Necrosis Factor-α
TOS: Total Oxidant Status
