# Supplementary material for: Co-administration of Naringin and NLRP3 Inhibitor Improves Myelin Repair and Mitigates Oxidative Stress in Cuprizone-Induced Demyelination Model
Source: Curr Neuropharmacol. 2024 Dec 6;23(4):475–91. doi: 10.2174/1570159X23666241206102022 (PMC12105273; doi:10.2174/1570159X23666241206102022)
Supplement: Supplementary file 1 [file CN-23-4-475_SD1.pdf]

Supplementary Material

Co-administration of Naringin and NLRP3 Inhibitor Improves Myelin Repair and Mitigates Oxidative Stress in Cuprizone-Induced Demyelination Model

Fatemeh Kalaki-Jouybari[1,2], Moein Shirzad[1,2], Mohammad Javan[3], Maryam Ghasemi-Kasman[4,5,*] and Mehdi Pouramir[2,4,*]

[1]Student Research Committee, Babol University of Medical Sciences, Babol, Iran; [2]Department of Clinical Biochemistry, Faculty of Medicine, Babol University of Medical Sciences, Babol, Iran; [3]Department of Physiology, Faculty of Medical Sciences, Tarbiat Modares University, Tehran, Iran; [4]Cellular and Molecular Biology Research Center, Health Research Institute, Babol University of Medical Sciences, Babol, Iran; [5]Department of Physiology, Faculty of Medicine, Babol University of Medical Sciences, Babol, Iran

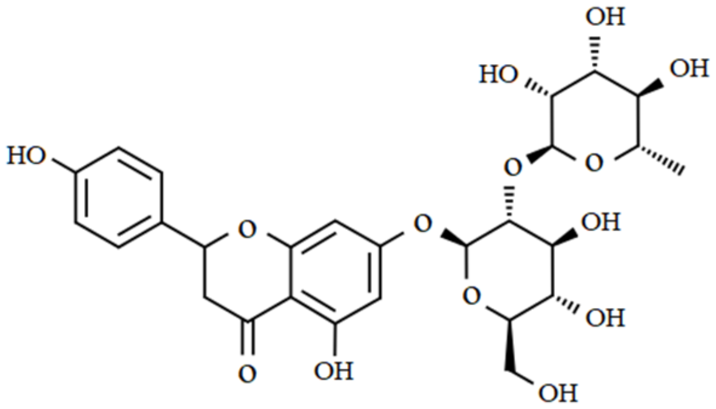

Fig. (S1). The chemical structure of naringin.

1875-6190/25 $65.00+.00 © 2025 Bentham Science Publishers
